# Evaluation of relationship between ruminal pH and the proportion of de novo fatty acids in milk

**DOI:** 10.3168/jdsc.2020-0042

**Published:** 2021-04-05

**Authors:** R. Fukumori, W. Shi, S. Oikawa, M. Oba

**Affiliations:** 1 Department of Veterinary Medicine, School of Veterinary Medicine, Rakuno Gakuen University, Ebetsu, Japan 069-8501; 2 Department of Agricultural, Food and Nutritional Science, University of Alberta, Edmonton, AB, Canada T6G 2P5

## Abstract

The objective of this study was to evaluate the relationship between ruminal pH and milk de novo fatty acid (DNFA) concentrations determined by Fourier-transform infrared spectroscopy. Data were collected from 18 multiparous Holstein cows fitted with a rumen cannula and fed 1 of the experimental diets differing in starch content (22.1 vs. 28.3%) with or without supplementation of a *Saccharomyces cerevisiae* fermentation product in a previous study. Milk was sampled on d 7 and 21 after calving, and concentrations of milk fat, DNFA (C6 to C14), mixed-origin fatty acids (FA; C16:0 and C16:1), and preformed FA (≥C18) were estimated using Fourier-transform infrared spectrometry. Ruminal pH was recorded in the ventral sac every 30 s continuously for 72 h on d 7 to 9 and 21 to 23 after calving. Daily maximum, nadir, and mean ruminal pH as well as duration and area below pH 5.8 were determined for each period. Milk DNFA (g/100 g of FA) was positively related to nadir (r = 0.428) and mean (r = 0.471) ruminal pH and negatively related to duration (r = −0.511) and area (r = −0.520) below pH 5.8. Milk fat content did not have a relationship with ruminal pH variables in this study. The regression lines for d 7 and 21 were similar, likely because plasma free FA concentrations were not different between d 7 and 21 (513 vs. 534 µEq/L) for the current data set. The coefficients of determination between DNFA and ruminal pH were greater for DNFA in total milk FA (g/100 g of FA) than in milk (g/100 g of milk), suggesting that DNFA in milk fat (g/100 g of FA) is an appropriate measurement variable that relates to ruminal pH even for cows in early lactation.

## Highlights

• Milk de novo fatty acids (DNFA) estimated by FTIR were correlated with ruminal pH.
• Ruminal pH was correlated better with DNFA than milk fat content or yield.
• Ruminal pH was correlated better with DNFA in milk fat than with DNFA in milk.

## Graphical Abstract

**Figure:**
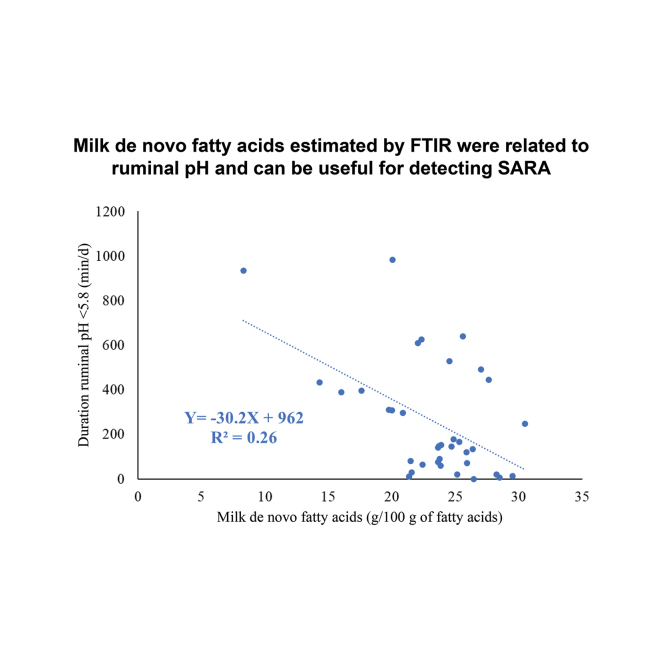
**Summary:** Subacute ruminal acidosis (SARA) is a serious problem in high-producing dairy cows, but its diagnosis is difficult. Recently, Fourier-transform infrared spectrometry (FTIR) was introduced to the dairy industry as a tool to estimate milk fatty acids (FA), such as de novo, mixed-origin, and preformed FA. The present study showed that milk de novo FA (g/100 g of FA) concentration was positively related to nadir and mean ruminal pH and negatively related to duration below pH 5.8. Milk fat content was not related to ruminal pH, indicating that milk de novo FA concentration is an appropriate measurement variable that relates to ruminal pH.

Subacute ruminal acidosis is a serious problem in high-producing dairy cows, but its diagnosis is difficult. The most accurate technique to detect cows with SARA is direct and continuous measurement of ruminal pH using an indwelling pH measurement system (Penner et al., 2007), but this may not be a useful method for on-farm diagnosis. Recently, analysis of milk fatty acid (**FA**) profiles such as de novo, mixed-origin, and preformed FA using Fourier-transform infrared spectrometry (**FTIR**) was introduced to the dairy industry as a management tool. De novo FA (**DNFA**; C4 to C14) are FA synthesized in the mammary gland using acetate and BHB derived from rumen fermentation or liver metabolism. Preformed FA (≥C18) derived from diet or adipose tissues enter the mammary gland from the blood stream. Mixed-origin FA (C16:0 and C16:1) are either DNFA or preformed FA. Depression of ruminal pH caused by consumption of diets high in rapidly fermentable carbohydrates or deficient in physically effective fiber affects biohydrogenation of dietary FA, increases CLA synthesis and absorption, and may reduce DNFA synthesis in the mammary gland (Bauman and Griinari, 2001). Woolpert et al. (2017) categorized dairy farms as having either high or low DNFA in their bulk milk and reported that farms with low DNFA had less bunk space, greater stocking density, and less feeding frequency, indicating that management to reduce the risk of SARA can contribute to greater milk DNFA. These findings also suggest that milk DNFA may be related to ruminal pH. However, there is little research data showing the relationship between milk DNFA and actual ruminal pH measured by a continuous pH measurement system. The objective of this study was to evaluate the relationship between ruminal pH and milk DNFA.

All experimental procedures were approved by the University of Alberta Animal Care and Use Committee for Livestock (AUP#1915) and conducted according to the guidelines of the Canadian Council on Animal Care (2009). Data were collected from 18 lactating Holstein cows fitted with a rumen cannula in the study of Shi et al. (2019). Cows were fed 1 of the experimental diets differing in starch content (22.1 or 28.3%) with or without supplementation of a *Saccharomyces cerevisiae* fermentation product (NutriTek, Diamond V Inc.). On d 7 ± 3 and 21 ± 3 after calving, milk samples were collected from 2 consecutive milkings (p.m. and a.m.). Milk samples were frozen in a freezer and kept at −20°C until analysis. Ruminal pH was recorded as described by Shi et al. (2019). Briefly, ruminal pH was continuously recorded in the ventral sac every 30 s for 72 h on d 7 to 9 ± 3 and 21 to 23 ± 3 after calving using an indwelling ruminal pH measurement system (LRCpH, Dascor) developed by Penner et al. (2007). Maximum, nadir, and mean ruminal pH as well as duration and area below pH 5.8 were determined daily for each cow and averaged for each period. The threshold pH 5.8 was used because ruminal pH lower than 5.8 depresses fibrolytic activity in the rumen (Russell and Wilson, 1996). Concentrations of milk fat, DNFA, mixed-origin FA, and preformed FA were predicted using a CombiScope FTIR A600 HP Dairy Analyzer (Delta Instruments) at the Kirishima milk testing laboratory (Miyazaki, Japan). Milk samples were thawed in a water bath at 41°C for 15 min, and all samples were measured at the same time. Milk DNFA, mixed-origin FA, and preformed FA were measured as grams per 100 g of milk by FTIR using partial least squares chemometric prediction models developed by Delta Instruments (parameter no. 9703 for absolute de novo, 9704 for absolute mixed, and 9705 for absolute preformed). In addition, proportions of DNFA, mixed-origin FA, and preformed FA in total FA (g/100 g of total FA) were calculated. The calibration of FTIR to estimate FA group was done at the time of factory shipment. The calibration to estimate fat concentration was done monthly using a calibration sample provided by Japan Dairy Technical Association. The reference chemistry value of fat concentration was determined using the Gerber method (AOAC International, 2000). No outliers were identified in the current data set; therefore, Pearson correlation coefficients were used to determine the relationship between ruminal pH variables and milk variables using JMP (version 13; SAS Institute Inc.). Significance was declared at *P* < 0.05. For variables with a significant correlation, slope, intercept, and coefficient of determination of the regression line were determined using the Fit Y by X procedure of JMP.

In the present study, neither milk fat content (%) nor yield (kg/d) were related to ruminal pH variables (r ≤ 0.137, *P* > 0.05; Table 1). Subacute ruminal acidosis is associated with reduced milk fat content (Allen, 1997). However, the present study failed to observe a relationship between milk fat content (%) and ruminal pH variables. Enemark et al. (2004) reported no correlation between ruminal pH and milk fat content (r = −0.060, *P* = 0.81) for cows in early lactation (DIM <30) but a tendency of positive correlation (r = 0.305, *P* = 0.059) for cows in mid lactation (DIM >30). In a meta-analysis by Allen (1997), a strong positive relationship between mean ruminal pH and milk fat content (r = 0.624, *P* < 0.01) was reported, but the analysis did not include early-lactation cows (approximate DIM <30). In early-lactation cows, excessive body fat mobilization contributes to total milk fat production (Bell, 1995), which likely makes milk fat content less sensitive to changes in ruminal pH. Therefore, milk fat content may not be a good predictor of ruminal pH, particularly for cows in early lactation.

**Table 1:** Regression equations and Pearson correlation coefficients between ruminal pH and milk composition on d 7 and 21 after calving^1^

Ruminal pH^2^	Item	De novo FA		Mixed-origin FA		Preformed FA		Fat	
		g/100 g of FA	g/100 g of milk	g/100 g of FA	g/100 g of milk	g/100 g of FA	g/100 g of milk	g/100 g of milk	kg/d
Maximum	r	0.287	0.270	0.208	0.202	0.262	−0.045	0.137	0.012
	Eq^3^	—	—	—	—	—	—	—	—
Nadir	r	0.428**	0.208	0.164	0.006	−0.332	−0.250	−0.077	−0.228
	Eq	Y = 0.021X + 5.0	—	—	—	—	—	—	—
Mean	r	0.471**	0.335*	0.391*	0.222	−0.458**	0.212	0.078	−0.091
	Eq	Y = 0.023X + 5.6	Y = 0.281X + 5.9	Y = 0.022X + 5.5	—	Y = −0.012X + 6.7	—	—	—
Duration	r	−0.511**	−0.354*	0.426**	−0.231	0.497**	0.262	−0.056	0.040
	Eq	Y = −30.2X + 962	Y = −366X + 538	Y = −28.9X + 1,109	—	Y = 16.7X − 530	—	—	—
Area	r	−0.520**	−0.343*	−0.433**	−0.213	0.506**	0.311	−0.022	0.143
	Eq	Y = −8.06X + 240	Y = −92.8X + 124	Y = −7.69X + 279	—	Y = 4.44X − 157	—	—	—

1 FA = fatty acids. De novo FA: C4 to C14; mixed-origin FA: C16:0 and C16:1; preformed FA: ≥C18.

2 Duration = duration below ruminal pH 5.8 (min/d). Area = area below ruminal pH 5.8 (pH·min/d).

3 Regression equation. Y = ruminal pH variables; X = milk variables.

* *P* < 0.05

** *P* < 0.01.

In the current study, milk DNFA (g/100 g of milk) was positively related to mean ruminal pH (r = 0.335, *P* < 0.05) and negatively related to duration (r = −0.354, *P* < 0.05) and area below pH 5.8 (r = −0.343, *P* < 0.05; Table 1). These findings are consistent with previous studies reporting that low ruminal pH decreases DNFA synthesis (Martel et al., 2011; Baumann et al., 2016). Under low ruminal pH, rumen bacteria alter the pathway of biohydrogenation of PUFA and shift to produce more *trans*-10,*cis*-12 CLA (Choi et al., 2005), which reduces yields of all milk FA and particularly DNFA synthesis in the mammary gland to a greater extent (Bauman and Griinari, 2001). Milk DNFA in total FA (g/100 g of FA) also showed correlations with ruminal pH variables. Milk DNFA (g/100 g of FA) was positively related to nadir (r = 0.428, *P* < 0.01) and mean ruminal pH (r = 0.471, *P* < 0.01) and negatively related to duration (r = −0.511, *P* < 0.01) and area below pH 5.8 (r = −0.520, *P* < 0.01). Interestingly, we found that the coefficients of determination between DNFA and ruminal pH variables were greater for DNFA in total milk FA (g/100 g of FA) than in milk (g/100 g of milk; mean pH: R^2^ = 0.222 vs. 0.112; duration <pH 5.8: R^2^ = 0.261 vs. 0.125; area <pH 5.8: R^2^ = 0.270 vs. 0.117). These data suggest that milk fat DNFA (g/100 g of FA) would be an appropriate measurement to predict ruminal pH rather than milk DNFA (g/100 g of milk), even for cows in early lactation.

Milk preformed FA (g/100 g of FA) was also related to mean pH (r = −0.458, *P* < 0.01), duration (r = 0.497, *P* < 0.01), and area below pH 5.8 (r = 0.506, *P* < 0.01; Table 1). In the present study, BW change after calving was positively related to mean ruminal pH (r = 0.434; *P* < 0.10) but negatively related to milk preformed FA (g/100 g of FA; r = −0.523, *P* < 0.05), suggesting that greater BW loss, which was associated with lower rumen pH, may have increased preformed FA in milk.

Subacute ruminal acidosis is characterized by duration of depressed ruminal pH (Plaizier et al., 2008). In addition, the area below pH 5.8 is considered to be an indicator of severity of SARA because it accounts for both duration and extent of ruminal pH depression (Penner et al., 2007). In accordance with these reports, the coefficients of determination between milk DNFA (g/100 g of FA) and duration (R^2^ = 0.261) or area below pH 5.8 (R^2^ = 0.270) were greater than those between milk DNFA and nadir (R^2^ = 0.183) or mean pH (R^2^ = 0.222), suggesting that the duration and severity of SARA are more important factors to reflect the ruminal environment and inhibit FA synthesis in the mammary gland.

The regression equation between milk DNFA (g/100 g of FA) and duration below ruminal pH 5.8 was developed using the pooled data from cows on d 7 and 21 after calving because regression lines for d 7 and 21 were similar (Figure 1), likely because plasma free FA concentrations were not different between d 7 and 21 (513 and 534 µEq/L, respectively; *P* = 0.74) for the current data set. Plasma free FA concentration reflects the extent of fat mobilization, and greater plasma free FA concentration may increase preformed FA in milk and decrease the proportion of milk DNFA (g/100 g of FA). As such, the regression equation between milk DNFA (g/100 g of FA) and duration below ruminal pH 5.8 might be different for cows in later stages of lactation, where plasma free FA concentrations are lower.

**Figure 1:**
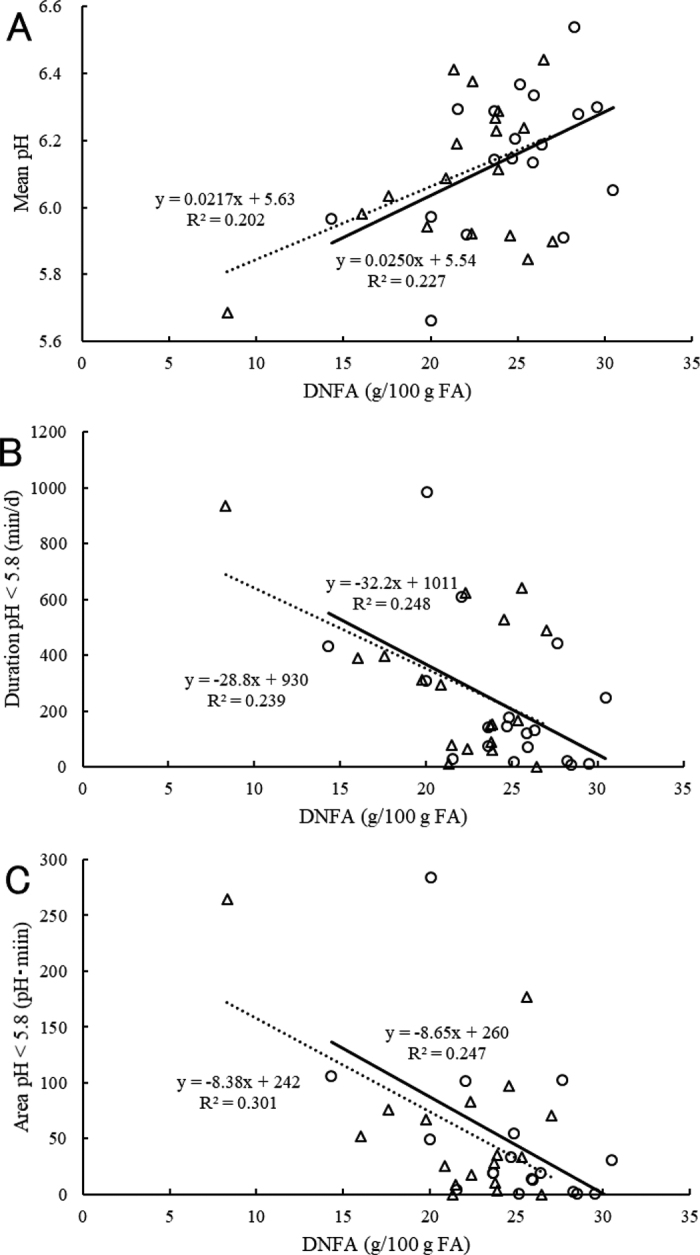
The relationship between milk de novo fatty acids (DNFA; g/100 g of FA) and mean pH (A), duration (B), and area below pH 5.8 (C) on d 7 (plot = triangle, regression line = dotted line, n = 18) and 21 (plot = circle, regression line = solid line, n = 18) after calving.

Previous research suggests that milk FA data from infrared spectrometry are useful as an alert for health problems or inappropriate nutritional management. Pape et al. (2018) showed that the onset of clinical ketosis and displaced abomasum was related to changes in milk variables, including DNFA concentrations, several days before clinical signs were visible. In addition, Bach et al. (2019) reported that cows with milk fat DNFA concentrations below 21.1 g/100 g of FA during 13 to 18 DIM had a 7.2-fold greater risk of a disease or removal event (cull or death) within 30 DIM compared with higher DNFA cows. For bulk tank milk, Woolpert et al. (2017) divided commercial dairy farms into 2 groups by their bulk tank milk DNFA concentration (high: mean = 24.6 g/100 g of FA; low: mean = 23.1 g/100 g of FA) and reported that feeding management to reduce the risk of SARA (e.g., frequency of feed delivery and push up, increased feed bunk space, and adequate dietary physically effective fiber) was related to greater milk DNFA concentrations. However, they did not measure actual ruminal pH. At commercial dairies, SARA is difficult to diagnosis because cows do not exhibit obvious signs. To the best of our knowledge, the current study is the first to show that milk DNFA concentration, estimated by infrared spectrometry, is related to ruminal pH, suggesting that milk DNFA concentration can indicate an occurrence of SARA. The DNFA concentrations presented in the current study should not be interpreted as absolute values because milk samples were frozen before analysis and calibration of DNFA was not conducted frequently enough. However, the current data would be useful to determine the relative relationship between ruminal pH variables and milk DNFA concentrations because the measurement by FTIR was done at the same time.

In conclusion, milk DNFA concentrations (g/100 g of FA), determined by FTIR, were related to ruminal pH variables and can be used as a rapid, noninvasive tool to detect SARA at commercial dairies. However, the relationships were not extremely strong, and further research with a larger number of samples is needed to determine a specific cut-off point for diagnosing SARA.
